# 1,4-Diazo­niabicyclo­[2.2.2]octane tetra­chloroiodate(III) chloride

**DOI:** 10.1107/S1600536810007865

**Published:** 2010-03-10

**Authors:** Li-Zhuang Chen

**Affiliations:** a School of Material Science and Engineering, Jiangsu University of Science and Technology, Zhenjiang 212003, People’s Republic of China

## Abstract

In the title compound, C_6_H_14_N_2_^2+^·Cl_4_I^−^·Cl^−^, the dication and the anions lie on special positions. The dication has *mm*2 symmetry with two bonded C atoms and the two N atoms located on a crystallographic mirror plane parallel to *bc*, and with a mirror plane parallel to *ab* passing through the mid points of the three C—C bonds. In the square-planar Cl_4_I^−^ anion, two Cl atoms and the I atom are located on the *mm*2 axis; the other two Cl atoms are disordered over two postions of equal occupancy (0.25) across the mirror parallel to the *ab* plane. The Cl^−^ anion is located on the *mm*2 axis. The crystal structure is stabilized by inter­molecular N—H⋯Cl hydrogen bonds.

## Related literature

For ferroelectric materials, see: Scott (2007 ▶); Katrusiak & Szafrański (2006 ▶).
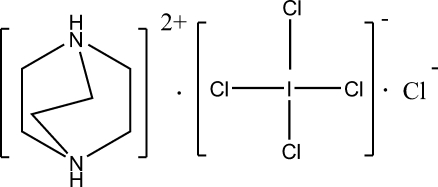


## Experimental

### 

#### Crystal data

C_6_H_14_N_2_^2+^·Cl_4_I^−^·Cl^−^
*M* *_r_* = 418.34
Orthorhombic, 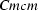

*a* = 8.1496 (16) Å
*b* = 21.904 (4) Å
*c* = 7.7184 (15) Å
*V* = 1377.8 (5) Å^3^
*Z* = 4
Mo *K*α radiation
μ = 3.26 mm^−1^
*T* = 293 K
0.28 × 0.25 × 0.20 mm

#### Data collection

Rigaku SCXmini diffractometer
Absorption correction: multi-scan (*CrystalClear*; Rigaku, 2005 ▶) *T*_min_ = 0.85, *T*_max_ = 0.90
7175 measured reflections
908 independent reflections
882 reflections with *I* > 2σ(*I*)
*R*_int_ = 0.028

#### Refinement

*R*[*F* ^2^ > 2σ(*F* ^2^)] = 0.019
*wR*(*F* ^2^) = 0.045
*S* = 1.25
908 reflections
48 parameters
H-atom parameters constrained
Δρ_max_ = 0.44 e Å^−3^
Δρ_min_ = −0.41 e Å^−3^

### 

Data collection: *CrystalClear* (Rigaku, 2005 ▶); cell refinement: *CrystalClear*; data reduction: *CrystalClear*; program(s) used to solve structure: *SHELXS97* (Sheldrick, 2008 ▶); program(s) used to refine structure: *SHELXL97* (Sheldrick, 2008 ▶); molecular graphics: *SHELXTL* (Sheldrick, 2008 ▶); software used to prepare material for publication: *SHELXL97*.

## Supplementary Material

Crystal structure: contains datablocks I, global. DOI: 10.1107/S1600536810007865/pv2257sup1.cif

Structure factors: contains datablocks I. DOI: 10.1107/S1600536810007865/pv2257Isup2.hkl

Additional supplementary materials:  crystallographic information; 3D view; checkCIF report

## Figures and Tables

**Table 1:** Hydrogen-bond geometry (Å, °)

*D*—H⋯*A*	*D*—H	H⋯*A*	*D*⋯*A*	*D*—H⋯*A*
N1—H1⋯Cl4^i^	0.91	2.29	3.028 (2)	138

Symmetry code: (i) 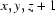.

**Fig. 1.**
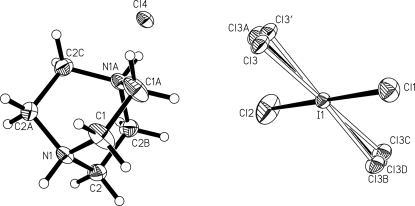
The title compound with atomic labels; displacement ellipsoids were drawn at the 30% probability level.

## supplementary crystallographic information

### Comment

Ferroelectric materials continue to attract much attention due to their potential applications in memory devices (Scott, 2007). Recently, diazabicyclo[2.2.2]octane (dabco) salts with inorganic tetrahedral anions having potassium dihydrophosphate-type (KDP-type) structures have been found to exhibit exceptional dielectric properties (Katrusiak & Szafrański, 2006). In our laboratory, the title compound containing a diprotonated cation, C_6_H_14_N_2_^2+^, a tetrachloroiodate and a Cl^-^ anions, has been synthesized. In this article, the crystal structure of the title compound is reported.

In the title compound (Fig. 1), all the species lie on special positions with only one quarter of each being part of the asymmetric unit. The I(III) ion in a square-planar coordination environment. The Cl3 atom is disordered. The crystal structure is stabilized by intermolecular N—H···Cl hydrogen bonds (Table 1).

### Experimental

KI (0.5 g) and I_2_ (0.7 g) were dissolved in a solution of ethanol (30 ml) and conc. HCl (13 ml) (36%). After addition of 1,4-diazoniabicyclo[2.2.2]octane (1 g) to the above solution, the mixture was stirred for 1 h and then filtered. The filtrate was left at room temperature to allow the solvent to evaporate. Yellow transparent block crystals were obtained after one weeks.

### Refinement

All H atoms were placed in calculated positions with C—H = 0.97 Å and N—H = 0.91 Å, and refined using a riding model, with U_iso_(H) = 1.2U_eq_(C/N). The Cl3 atom was disordered over two sites

### Crystal data

**Table:**

C_6_H_14_N_2_^2+^·Cl_4_I^−^·Cl^−^	*F*(000) = 808
*M**_r_* = 418.34	*D*_x_ = 2.017 Mg m^−^^3^
Orthorhombic, *C**m**c**m*	Mo *K*α radiation, λ = 0.71073 Å
Hall symbol: -C 2c 2	Cell parameters from 882 reflections
*a* = 8.1496 (16) Å	θ = 3.2–27.5°
*b* = 21.904 (4) Å	µ = 3.26 mm^−^^1^
*c* = 7.7184 (15) Å	*T* = 293 K
*V* = 1377.8 (5) Å^3^	Block, yellow
*Z* = 4	0.28 × 0.25 × 0.20 mm

### Data collection

**Table:**

Rigaku SCXmini diffractometer	908 independent reflections
Radiation source: fine-focus sealed tube	882 reflections with *I* > 2σ(*I*)
graphite	*R*_int_ = 0.028
Detector resolution: 13.6612 pixels mm^-1^	θ_max_ = 27.5°, θ_min_ = 3.2°
ω scans	*h* = −10→10
Absorption correction: multi-scan (*CrystalClear*; Rigaku, 2005)	*k* = −28→27
*T*_min_ = 0.85, *T*_max_ = 0.90	*l* = −9→10
7175 measured reflections	

### Refinement

**Table:**

Refinement on *F*^2^	Secondary atom site location: difference Fourier map
Least-squares matrix: full	Hydrogen site location: inferred from neighbouring sites
*R*[*F*^2^ > 2σ(*F*^2^)] = 0.019	H-atom parameters constrained
*wR*(*F*^2^) = 0.045	*w* = 1/[σ^2^(*F*_o_^2^) + (0.0179*P*)^2^ + 1.0795*P*] where *P* = (*F*_o_^2^ + 2*F*_c_^2^)/3
*S* = 1.25	(Δ/σ)_max_ < 0.001
908 reflections	Δρ_max_ = 0.44 e Å^−^^3^
48 parameters	Δρ_min_ = −0.41 e Å^−^^3^
0 restraints	Extinction correction: *SHELXL97* (Sheldrick, 2008), Fc^*^=kFc[1+0.001xFc^2^λ^3^/sin(2θ)]^-1/4^
Primary atom site location: structure-invariant direct methods	Extinction coefficient: 0.0042 (2)

### Special details

**Table:**

Geometry. All esds (except the esd in the dihedral angle between two l.s. planes) are estimated using the full covariance matrix. The cell esds are taken into account individually in the estimation of esds in distances, angles and torsion angles; correlations between esds in cell parameters are only used when they are defined by crystal symmetry. An approximate (isotropic) treatment of cell esds is used for estimating esds involving l.s. planes.
Refinement. Refinement of *F*^2^ against ALL reflections. The weighted *R*-factor wR and goodness of fit *S* are based on *F*^2^, conventional *R*-factors *R* are based on *F*, with *F* set to zero for negative *F*^2^. The threshold expression of *F*^2^ > σ(*F*^2^) is used only for calculating *R*-factors(gt) etc. and is not relevant to the choice of reflections for refinement. *R*-factors based on *F*^2^ are statistically about twice as large as those based on *F*, and *R*- factors based on ALL data will be even larger.

### Fractional atomic coordinates and isotropic or equivalent isotropic displacement parameters (Å^2^)

**Table:**

	*x*	*y*	*z*	*U*_iso_*/*U*_eq_	Occ. (<1)
N1	0.0000	0.34601 (10)	0.9105 (3)	0.0342 (5)	
H1	0.0000	0.3460	1.0284	0.041*	
C1	0.0000	0.41040 (14)	0.8486 (4)	0.0590 (10)	
H1A	0.0965	0.4315	0.8915	0.071*	0.50
H1B	−0.0965	0.4315	0.8915	0.071*	0.50
C2	0.1498 (3)	0.31364 (11)	0.8488 (3)	0.0442 (5)	
H2A	0.1502	0.2720	0.8917	0.053*	
H2B	0.2472	0.3341	0.8917	0.053*	
I1	0.5000	0.453833 (11)	0.2500	0.03008 (11)	
Cl1	0.5000	0.56970 (5)	0.2500	0.0529 (3)	
Cl2	0.5000	0.34147 (6)	0.2500	0.0918 (6)	
Cl3	0.1864 (17)	0.4465 (8)	0.2500	0.0442 (7)	0.50
Cl3'	0.2037 (18)	0.4541 (8)	0.223 (2)	0.0442 (7)	0.25
Cl4	0.0000	0.27673 (5)	0.2500	0.0396 (2)	

### Atomic displacement parameters (Å^2^)

**Table:**

	*U*^11^	*U*^22^	*U*^33^	*U*^12^	*U*^13^	*U*^23^
N1	0.0441 (13)	0.0360 (12)	0.0226 (11)	0.000	0.000	0.0025 (9)
C1	0.109 (3)	0.0320 (15)	0.0356 (17)	0.000	0.000	−0.0016 (13)
C2	0.0339 (11)	0.0605 (14)	0.0381 (12)	0.0051 (10)	−0.0025 (9)	0.0047 (10)
I1	0.02875 (15)	0.03053 (15)	0.03098 (15)	0.000	0.000	0.000
Cl1	0.0619 (8)	0.0343 (5)	0.0624 (7)	0.000	0.000	0.000
Cl2	0.0792 (11)	0.0290 (6)	0.167 (2)	0.000	0.000	0.000
Cl3	0.025 (2)	0.055 (3)	0.052 (4)	−0.005 (3)	0.000	0.000
Cl3'	0.025 (2)	0.055 (3)	0.052 (4)	−0.005 (3)	0.000	0.000
Cl4	0.0516 (6)	0.0410 (5)	0.0261 (4)	0.000	0.000	0.000

### Geometric parameters (Å, °)

**Table:**

N1—C1	1.489 (4)	I1—Cl3'	2.424 (16)
N1—C2	1.490 (2)	I1—Cl3'^iii^	2.424 (16)
N1—C2^i^	1.490 (2)	I1—Cl3'^iv^	2.424 (16)
N1—H1	0.9100	I1—Cl3'^v^	2.424 (16)
C1—C1^ii^	1.522 (7)	I1—Cl2	2.4612 (14)
C1—H1A	0.9700	I1—Cl1	2.5379 (13)
C1—H1B	0.9700	I1—Cl3	2.561 (15)
C2—C2^ii^	1.525 (4)	I1—Cl3^iv^	2.561 (15)
C2—H2A	0.9700	Cl3'—Cl3'^v^	0.42 (4)
C2—H2B	0.9700		
			
C1—N1—C2	110.37 (15)	Cl3'^iv^—I1—Cl3'^v^	179.7 (8)
C1—N1—C2^i^	110.37 (15)	Cl3'—I1—Cl2	90.2 (4)
C2—N1—C2^i^	110.0 (2)	Cl3'^iii^—I1—Cl2	90.2 (4)
C1—N1—H1	108.7	Cl3'^iv^—I1—Cl2	90.2 (4)
C2—N1—H1	108.7	Cl3'^v^—I1—Cl2	90.2 (4)
C2^i^—N1—H1	108.7	Cl3'—I1—Cl1	89.8 (4)
N1—C1—C1^ii^	108.72 (16)	Cl3'^iii^—I1—Cl1	89.8 (4)
N1—C1—H1A	109.9	Cl3'^iv^—I1—Cl1	89.8 (4)
C1^ii^—C1—H1A	109.9	Cl3'^v^—I1—Cl1	89.8 (4)
N1—C1—H1B	109.9	Cl2—I1—Cl1	180.0
C1^ii^—C1—H1B	109.9	Cl3'^iii^—I1—Cl3	173.9 (4)
H1A—C1—H1B	108.3	Cl3'^iv^—I1—Cl3	173.9 (4)
N1—C2—C2^ii^	108.65 (12)	Cl2—I1—Cl3	86.4 (4)
N1—C2—H2A	110.0	Cl1—I1—Cl3	93.6 (4)
C2^ii^—C2—H2A	110.0	Cl3'—I1—Cl3^iv^	173.9 (4)
N1—C2—H2B	110.0	Cl3'^v^—I1—Cl3^iv^	173.9 (4)
C2^ii^—C2—H2B	110.0	Cl2—I1—Cl3^iv^	86.4 (4)
H2A—C2—H2B	108.3	Cl1—I1—Cl3^iv^	93.6 (4)
Cl3'—I1—Cl3'^iii^	179.7 (9)	Cl3—I1—Cl3^iv^	172.8 (7)
Cl3'—I1—Cl3'^iv^	170.0 (8)	Cl3'^v^—Cl3'—I1	85.0 (4)
Cl3'^iii^—I1—Cl3'^v^	170.0 (8)		

Symmetry codes: (i) −*x*, *y*, *z*; (ii) *x*, *y*, −*z*+3/2; (iii) −*x*+1, *y*, −*z*+1/2; (iv) −*x*+1, *y*, *z*; (v) *x*, *y*, −*z*+1/2.

### Hydrogen-bond geometry (Å, °)

**Table:**

*D*—H···*A*	*D*—H	H···*A*	*D*···*A*	*D*—H···*A*
N1—H1···Cl4^vi^	0.91	2.29	3.028 (2)	138

Symmetry codes: (vi) *x*, *y*, *z*+1.
